# Linoleic Acid Promotes Emission of Bark Beetle Semiochemicals by Fungal Symbionts

**DOI:** 10.1007/s10886-022-01400-3

**Published:** 2022-12-30

**Authors:** C. Rikard Unelius, Suresh Ganji, Paal Krokene

**Affiliations:** 1 Department of Chemistry and Biomedical Sciences, Faculty of Health and Life Sciences, Linnaeus University, 391 82 Kalmar, Sweden; 2 Division of Biotechnology and Plant Health, Norwegian Institute of Bioeconomy Research, P.O. Box 115, 1431 Ås, Norway

**Keywords:** Tree-Killing Bark Beetles, Symbiont Fungi, Fatty Acid Composition, Linoleic Acid, Semiochemicals, Pheromones, Spiroacetals

## Abstract

Tree-killing bark beetles in conifer forests vector symbiotic fungi that are thought to help the beetles kill trees. Fungal symbionts emit diverse volatile blends that include bark beetle semiochemicals involved in mating and host localization. In this study, all 12 tested fungal isolates emitted beetle semiochemicals when growing in medium amended with linoleic acid. These semiochemicals included the spiroacetals chalcogran, *trans*-conophthorin and *exo-*brevicomin, as well as 2-methyl-3-buten-1-ol, the main aggregation pheromone component of the spruce bark beetle *Ips typographus*. The emission of these compounds was affected by the type of fatty acid present (linoleic vs. oleic acid). Accumulating evidence shows that the fatty acid composition in conifer bark can facilitate colonization by bark beetles and symbiotic fungi, whereas the fatty acid composition of non-host trees can be detrimental for beetle larvae or fungi. We hypothesize that beetles probe the fatty acid composition of potential host trees to test their suitability for beetle development and release of semiochemicals by symbiotic fungi.

**Supplementary Information:**

The online version contains supplementary material available at 10.1007/s10886-022-01400-3.

## Introduction

Some bark beetle species, such as the mountain pine beetle (*Dendroctonus ponderosae*) and the Eurasian spruce bark beetle (*Ips typographus*), are devastating tree-killers in coniferous forests in the Northern Hemisphere (Meddens et al. 2012; Wermelinger 2004). Most tree-killing bark beetles species vector symbiotic bluestain fungi that may help overwhelming tree defenses (Krokene 2015; Paine et al. 1997; Zhao et al. 2019). We and others have previously shown that these fungal symbionts emit a diverse bouquet of volatile compounds, some of which are pheromone components used by bark beetles in mating and host colonization (Cale et al. 2019; Kandasamy et al. 2019; Zhao et al. 2015). The emission of bark beetle pheromones by fungal symbionts appears to be an example of convergent evolution of semiochemicals across biological kingdoms (Zhao et al. 2019).

**Fig. 1:**
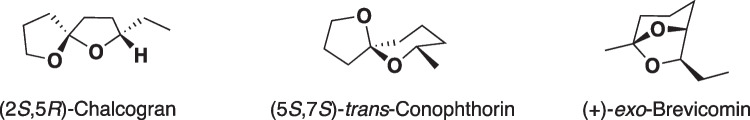
Spiroacetals that function as bark beetle semiochemicals and that have been identified in emissions from fungal symbionts of bark beetles

Chalcogran (IUPAC name: 2-ethyl-1,6-dioxaspiro[4,4]nonane; Fig. 1) is an aggregation pheromone for the six-spined bark beetle (*Pityogenes chalcographus*), a secondary pest in young Norway spruce [*Picea abies* (L.) Karst.] stands in Europe. In the 1970s, Wittko Francke and co-workers isolated chalcogran by treating 100,000 beetles of both sexes with a juvenile hormone analog that induced chalcogran synthesis in males (Francke et al. 1977). Both the *E*-and *Z*-stereoisomer of chalcogran have been identified from *P. chalcographus* and the *E*-isomer (2*S*,5*R*) (Fig. 1) is the behaviorally active stereoisomer (Byers et al. 1989).

*Trans*-conophthorin (IUPAC name: 7-methyl-1,6-dioxaspiro-4,5-decane [(*S,S*)-*t*C]; Fig. 1) is an anti-attractant or repellant for many bark beetle species, including *Ips typographus* (Unelius et al. 2014)*.* The natural occurring (*S,S*)-*t*C stereoisomer (Fig. 1) is physiologically active, while its antipode (*R,R*)-*t*C is not (Zhang et al. 2002).

Some bark beetle-associated fungi have also evolved the capability to biosynthesize the spiroacetal (1*R*,5*S*,7*R*)-( +)-*exo-*brevicomin (Zhao et al. 2019) (Fig. 1). This is an aggregation pheromone component for the western pine beetle (*Dendroctonus brevicomis*﻿) (Stewart et al. 1977; Wood et al. 1976) and the mountain pine beetle (Francke et al. 1996).

Bark beetles probably land on many potential host trees and probe the bark before they decide to enter or leave the tree (Paynter et al. 1990). Apparently, the beetles are probing for negative or positive cues that lead them to abort colonization or enter the host. Potential cues may be the level of defense chemicals in the bark or the composition of primary metabolites such as fatty acids. Fatty acids are aliphatic long-chain carboxylic acids. Fatty acid composition has been shown to facilitate colonization of jack pine (*Pinus banksiana*) bark by the mountain pine beetle (Ishangulyyeva et al. 2016). The fatty acid composition of a non-host deciduous tree was not suitable for the beetle larvae and Ishangulyyeva and co-workers concluded that fatty acid composition can be an important determinant of host suitability for bark beetles and their microbial symbionts.

In this study, we determine volatile emissions from several fungal symbionts of tree-killing bark beetles growing on agar media amended with two fatty acids: oleic acid ((9*Z*)-9-octadecenoic acid, C_18_H_34_O_2_) is a monounsaturated omega-9 fatty acid with a single double bond and linoleic acid ((9*Z*,12*Z*)-9,12-octadecadieneoic acid, C_18_H_32_O_2_) is a diunsaturated omega-6 fatty acid with two double bonds separated by a methylene group. Our choice of linoleic acid was inspired by a study identifying spiroacetal emissions from fungal spores growing in the presence of this fatty acid (Beck et al. 2012). Oleic acid was selected as a structurally similar control to test if promotion of spiroacetal emissions was specific for linoleic acid or required fatty acids in general. Oleic and linoleic acid are some of the most common fatty acids in nature and are important building blocks of the triglyceride constituents of cell membranes (Spector and Yorek 1985).

## Methods and Materials

### Fungal Isolates

We tested 12 isolates of bark beetle-associated fungi representing 10 species: *Ceratocystiopsis minuta* (isolate Cmin_1 and Cmin_2), *Endoconidiophora rufipennis* (Eruf), *Grosmannia clavigera* (Gcla)*, Grosmannia europhioides* (Geur)*, Leptographium abietinum* (Labi), *Ophiostoma ainoe* (Oain_1 and Oain_2), *Ophiostoma bicolor* (Obic), *Ophiostoma montium* (Omon), *Ophiostoma piceae* (Opic), and *Ophiostoma pseudotsugae* (Opse) (Supplementary Table S1). All isolates were obtained from the culture collection of the Norwegian Institute of Bioeconomy Research in Ås, Norway.

### Growth Media and Fungal Incubation

Fungal isolates were incubated on malt agar medium amended with the fatty acids oleic acid or linoleic acid. Three 500 mL glass bottles (A, B and C) were filled with growth medium consisting of 250 mL distilled water, 6 g Bacto agar (Nordic Biolabs), and 4.2 g LP 0039 Oxoid malt extract. Bottle A (control) contained pure growth medium, bottle B (oleic acid) was amended with 3 g oleic acid (Sigma-Aldrich, Sweden), and bottle C (linoleic acid) was amended with 3 g linoleic acid (Lancaster, USA). The media were autoclaved at 120 °C for 2 h, allowed to cool down to 60–70 °C, and poured into 100 mL E-flasks (10 mL per flask). The flasks were placed horizontally and when the medium had solidified fungus-colonized agar medium was placed in the center of each flask using disposable plastic inoculating loops, before the flasks were sealed with aluminum foil. Fatty acids are insoluble in water at room temperature but became soluble after autoclavation and formed a gel-like solid with the agar medium when it cooled down.

### Collection of Emitted Volatiles

To test how fatty acids affected fungal volatile emission, we incubated the 12 fungal isolates listed above on each of the three growth media (control, oleic acid, linoleic acid). Each isolate was incubated in three E-flasks and three flasks served as growth medium controls (*n* = 3, a total of 39 flasks per growth medium). The following day we punctured the aluminum foil seal of each flask with an SPME (solid phase microextraction) fiber holder and extracted the headspace in each flask using an SPME fiber. We used activated gray-colored SPME fibers 50/30 μM coated with divinylbenzene/-carboxen/polydimethylsiloxane (DVB/CAR/PDMS) and equipped with a 24 gauge needle (Supelco, Sigma-Aldrich, Stockholm, Sweden). The SPME adsorption time over the fungi was 35–40 min at room temperature for each sample. After sampling, the hole in the aluminum foil was sealed with a piece of tape. New headspace samples were collected at 3–4-day intervals from day 2 to day 30 after inoculation.

### GC–MS Analysis

The chemical composition of the headspace from each flask was determined by GC–MS (Gas Chromatography﻿–Mass Spectrometry). Each SPME fiber was kept in the GC–MS inlet (220 °C) for 5 min to clean it before sample absorption. The GC–MS instrument used was an Agilent 6890 GC (Santa Clara CA, USA) coupled with a Hewlett Packard 5973 MS mass detector (Palo Alto CA, USA). Helium was used as carrier gas (0.9 mL/min) on a polar enantioselective capillary column (Cyclosil B, 30 m × 0.25 mm, ID 0.25 µm stationary phase, J&W Scientific, USA). Mass spectra were obtained by electron impact ionization (70 eV). We used the following GC temperature program: initial temperature 40 °C (held for 3 min), raised to 150 °C at a rate of 3 °C min^−1^ and then to 250 °C at 15 °C min^−1^ (held for 10 min) (splitless). Volatiles were identified by injection of reference chemicals (see below). Relative semiochemical emissions were quantified by calculating the percentage of each chemical relative to total volatile emissions analyzed by SPME–GC–MS.

### Chemicals

All reference chemicals were obtained from commercial sources (Aldrich, Germany or Lancaster, USA) and used without further purification if not stated otherwise. For confirmation of *exo*-brevicomin, we synthesized all four stereoisomers of brevicomin for unambiguous identification of the stereochemistry of fungus-emitted compounds (Bohman et al. 2017; Page et al. 1986, 1990a, 1990b). The four stereoisomers of *trans*-conophthorin were obtained by transition metal-mediated hydrogenation of dehydroconophthorin (DHC). DHC was synthesized as reported by Unelius et al. (2014). The elution order was determined using a reference of (5*S*,7*S*)-trans-conophthorin obtained from Prof. W. Francke (University of Hamburg) via SLU, Alnarp, Sweden. For confirmation of chalcogran, all four stereoisomers were obtained from Erik Hedenström, MidSweden University, Sundsvall, Sweden (Högberg et al. 1987).

## Results

**Fig. 2:**
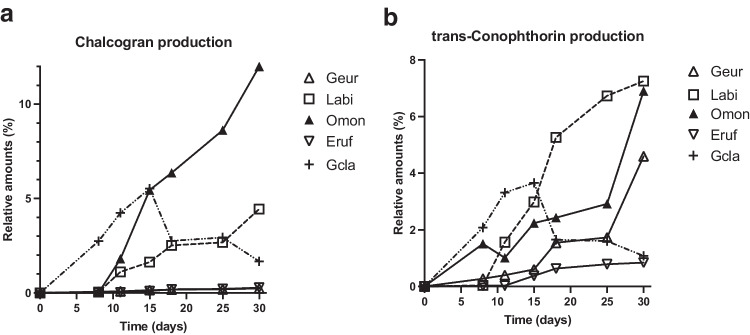
Emission of the bark beetle semiochemicals chalcogran and *trans*-conophthorin from linoleic acid-amended agar medium colonized by the bark beetle-associated fungi *Grosmannia europhioides* (Geur), *Leptographium abietinum* (Labi), *Ophiostoma montium* (Omon), *Endoconidiophora rufipennis* (Eruf), or *G. clavigera* (Gcla). Values shown are percentage of each bark beetle semiochemical relative to total volatile emissions analyzed by SPME–GC–MS.

**Fig. 3:**
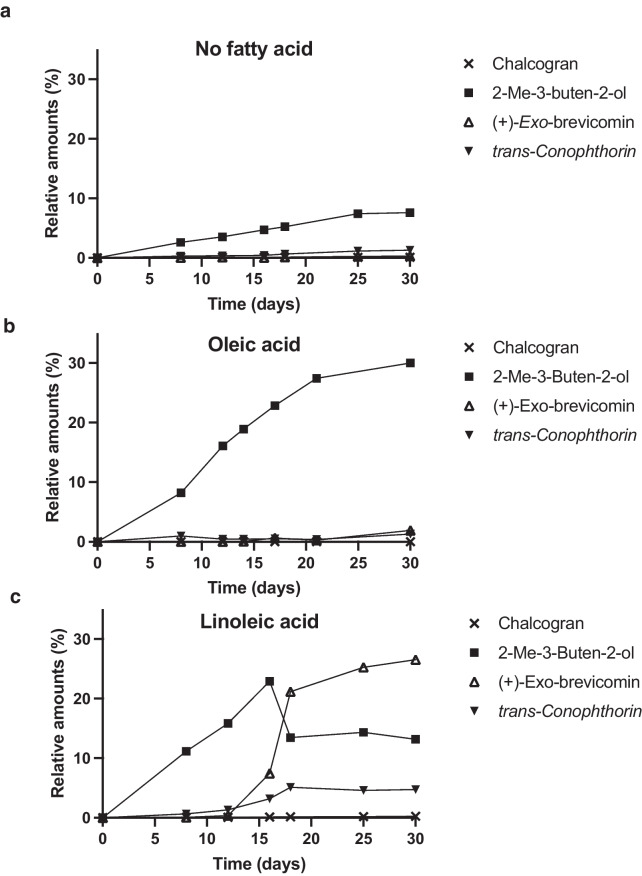
Emission of the bark beetle semiochemicals chalcogran, 2-methyl-3-buten-1-ol, ( +)-*exo*-brevicomin, and *trans*-conophthorin by *Grosmannia europhioides* growing in medium with different fatty acid content. Semiochemical emissions were measured at 3–7-day intervals and expressed as the percentage of each chemical relative to total volatile emissions analyzed by SPME–GC–MS

When growing in media amended with linoleic acid all 12 tested fungal isolates emitted chalcogran (after 10 days) and *trans*-conophthorin (after 8 days or less) (Fig. 2 and 3). All the tested fungi emitted all four chalcogran stereoisomers and both *trans*-conophthorin diastereomers when growing in the presence of linoleic acid.

We also analyzed spiroacetal emissions from fungi growing in media without any fatty acids or in media amended with oleic acid, a monounsaturated fatty acid which is otherwise similar to linoleic acid. Only two of the 12 examined bark beetle-associated fungi, *G. europhioides* (Fig. 3) and *L. abietinum,* emitted chalcogran or *trans*-conophthorin in medium with oleic acid or without any fatty acids, and then only in minor amounts (< 1% of total volatile emissions).

**Fig. 4:**
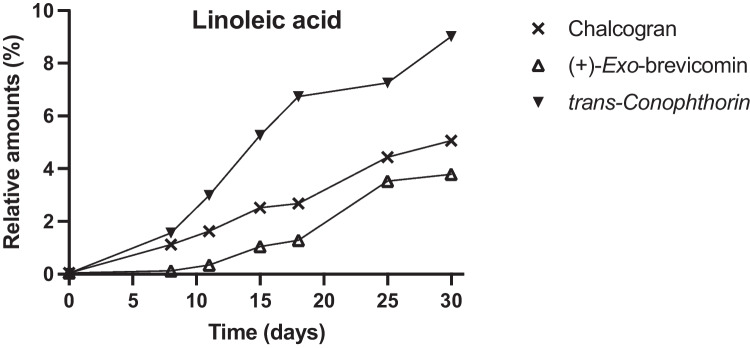
Spiroacetal emission by the fungus *Leptographium abietinum* incubated on malt agar medium amended with linoleic acid. Semiochemical emission was measured at 3–7-day intervals and expressed as the percentage of each chemical relative to total volatile emissions analyzed by SPME–GC–MS. No emission of spiroacetals was detected when *L. abietinum* was incubated on control medium or medium amended with oleic acid

*Grosmannia europhioides* and *L. abietinum* also emitted another spiroacetal, (1*R*,5*S*,7*R*)-( +)-*exo-*brevicomin (Fig. 3 and 4), an aggregation pheromone component for the tree-killing western pine beetle and mountain pine beetle. *Leptographium abietinum* only emitted ( +)-*exo-*brevicomin when growing in presence of linoleic acid, whereas *G. europhioides* also emitted ( +)-*exo-*brevicomin (in small amounts) when growing in medium with oleic acid or without any fatty acids (Fig. 3).

Both *L. abietinum* and *G. europhioides* stereoselectively emitted the (1*R*,5*S*,7*R*)-( +)-*exo-*brevicomin stereoisomer, *i.e.,* the same enantiomer as the two beetle species use as a pheromone component. Emission of ( +)-*exo*-brevicomin by *G. europhioides* could only be detected when the fungus was growing on medium amended with linoleic acid, whereas oleic acid did not mediate ( +)-*exo*-brevicomin emission (Fig. 3). *Grosmannia europhioides* also emitted two other bark beetle semiochemicals in the absence of linoleic acid (Fig. 3). These were 2-methyl-3-buten-2-ol, the main aggregation pheromone component for its vector *I. typographus* (Zhao et al. 2015), and minute amounts of (*S*,*S*)- *trans*-conophthorin, an anti-attractant for the same species (Unelius et al. 2014). *Trans*-conophthorin was emitted in higher relative proportions when linoleic acid was added to the medium. The mean value of the triplicate analysis of relative emission of 2-methyl-3-buten-2-ol was two- to three-fold higher in the presence of either oleic or linoleic acid than in medium with no fatty acids (Fig. 3).

When *L. abietinum* was incubated in media amended with linoleic acid we detected chalcogran and *trans*-conophthorin in the headspace, along with ( +)-*exo*-brevicomin (Fig. 4). *Leptographium abietinum* emitted a higher relative proportion of *trans*-conophthorin compared with *G. europhioides*.

### Relative Ratio of Chalcogran Stereoisomers

**Fig. 5:**
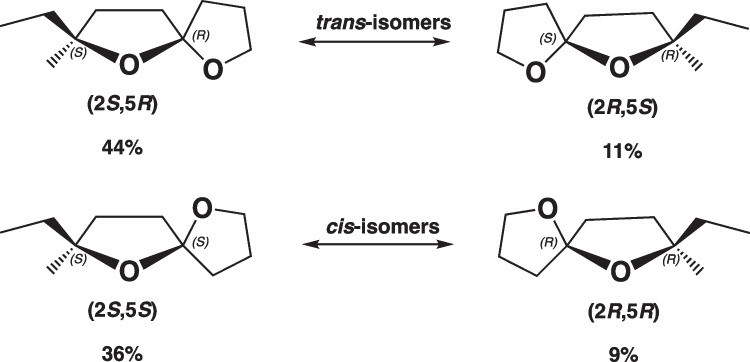
The ratio of all four chalcogran isomers detected in the headspace over the fungus *Grosmannia europhioides* incubated on malt agar medium amended with linoleic acid

**Fig. 6:**
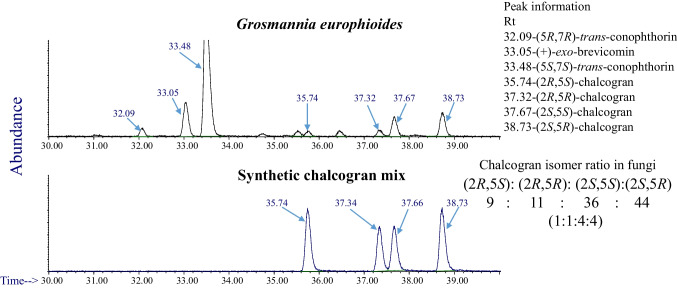
Enantioselective gas chromatography (Cyclodex B column) of pheromone components emitted by the fungus *Grosmannia europhioides* incubated on malt agar medium amended with linoleic acid (top) and of a synthetic mixture of chalcogran stereoisomers (bottom). Retention time for the different enantiomers is given next to the peaks. Retention order is in accordance with the literature (Trapp and Schurig 2001)

According to the literature, the (2*S*,5*R*)-chalcogran *trans*-diastereomer is the most behaviorally active pheromone enantiomer for *Pityogenes chalcographus* (Byers et al. 1989). In our SPME–GC–MS analyses of fungal headspaces we detected all four chalcogran stereoisomers, but the most behaviorally active stereoisomer was detected in larger amounts than the others (Fig. 5). For example, in headspace analyses of *G. europhioides* we detected the four chalcogran isomers in the ratio 9:11:36:44 (*RS:RR:SS:SR*) (Fig. 5 and 6). Thus, each (2*S*)-isomer (*SS, SR*) was detected at a four-fold higher ratio than the corresponding (2*R*)-isomers (*RS, RR*).

### Non-Acetal Metabolites Emitted in the Presence of Unsaturated Fatty Acids

When oleic acid or linoleic acid was added to the growth medium, several volatile metabolites in addition to spiroacetals was detected. These were oxidized products of linoleic and oleic acid, such as C5-C10 aldehydes, C3-C12 ketones, C4-C8 carboxylic acids, saturated and unsaturated esters of C2-C8 acids, and 2-alkyl branched furan derivatives from C2-C8 compounds. Most of these are known as products of enzymatic fungal metabolism, although some metabolites (e.g., 2-octenal) could have been formed by normal abiotic air oxidation. Among these compounds, 2-pentylfuran (35–57% of total volatile emissions), hexanal (12–40%), 2-octenal (5–9%), and hexanoic acid (5–13%) were detected in relatively large proportions. After 2–3 weeks incubation, only 2-pentylfuran could be detected and the emission of all other compounds had ceased.

## Discussion

Our results show that bark beetle-associated fungi emitted several compounds that are semiochemicals for bark beetles and other bark and wood-boring insects, such as longhorn beetles (Cerambycidae). Furthermore, emission was altered or enhanced when the fungi grew in the presence of linoleic acid. All the tested fungi emitted isomers of chalcogran and *trans*-conophthorin when linoleic acid was available in the growth medium. Although the emission of e.g. chalcogran was relatively modest it may still be ecologically relevant, as a release of only 1 mg per day attracts *P. chalcographus* to pheromone lures in the field (Byers 1993).

There are many proposed pathways for biosynthesis of the spiroacetals chalcogran, *trans-*conophthorin, and *exo*-brevicomin. However, isotope labelling studies suggests that the three compounds are biosynthesized via 3-hexen-1-ol (for *trans-*conophthorin and chalcogran) and 6-nonen-2-one (for brevicomins) (Song et al. 2014a; Song et al. 2014b; Tittiger and Blomquist 2017). In the postulated biosynthetic pathway from linoleic acid to *trans*-conophthorin (Beck et al. 2012), also 1-hexanol splits off. Precisely these two components (*trans*-conophthorin and 1-hexanol) were proven to be very active anti-attractants at low doses (0.05% for *trans*-conophthorin) in a field experiment with *I. typographus* (Unelius et al. 2014).

Our finding that bark beetle-associated fungi emitted chalcogran in the presence of linoleic acid corresponds well with the study by Beck and co-workers, who identified several spiroacetals in fungal spores growing in the presence of this fatty acid (Beck et al. 2012). The fact that linoleic acid, but not oleic acid, promoted spiroacetal biosynthesis in all the bark beetle-associated fungi we studied, suggests that these fungi biosynthesize chalcogran and other spiroacetals from linoleic acid. The proportion of chalcogran *trans* and *cis* diastereomers in thermodynamic equilibrium is 54% *trans* and 46% *cis* (Byers et al. 1989; Trapp and Schurig 2001). This corresponds closely with our results, where both the (2*R*)-pair and the (2*S*)-pair of stereoisomers had a 55:45 diastereomeric ratio (11:9 and 44:36). As the acetal stereocenter at carbon 5 in chalcogran is easily epimerized in non-alkaline solutions or at elevated temperatures, it is very likely that a reaction to the thermodynamic equilibrium between diastereomers occurred in the fungi.

Fungal biosynthesis of chalcogran from linoleic acid is further supported by our results showing that spiroacetal biosynthesis was specific to the kind of fatty acid added to the growth medium: addition of linoleic acid, but not oleic acid, promoted spiroacetal synthesis. The only structural difference between these two fatty acids is that linoleic acid has an additional homo-conjugated double bond. Thus, this double bond appears to play an essential role in fungal biosynthesis of spiroacetals.

The suggestion that the two double bonds in linoleic acid are necessary for fungal biosynthesis of spiroacetals, and the observation that oleic acid is not a suitable precursor for spiroacetal production, lead us to postulate a new hypothesis on the importance of fatty acid composition in conifer bark to bark beetles. We hypothesize that, due to the importance of fatty acids for the beetles’ fungal symbionts, fatty acid content acts as a cue for host acceptance by probing bark beetles. By selecting host trees with high levels of linoleic acid the beetles are more likely to get help from their fungal symbionts with pheromone production and release.

This hypothesis is supported by the results of Ishangulyyeva and co-workers who studied how fatty acid composition affected host suitability of jack pine (*Pinus banksiana*) for the mountain pine beetle and its symbiotic fungus *G. clavigera* (Ishangulyyeva et al. 2016). Both the beetle and the fungus thrived in artificial media amended with fatty acids at concentrations that are present in jack pine. In contrast, media with a fatty acid composition found in a non-host tree were less suitable for both. Ishangulyyeva et al. concluded that concentrations of individual fatty acids determined host suitability of jack pine and that a tree’s fatty acid profile might serve as an indicator and predictor for how suitable the tree is for colonization by bark beetles and fungi. Specifically, probing of a potential host tree by pioneer beetles, i.e., the first beetles to land on a tree before mass attack has been initiated, could involve evaluation of key fatty acids in the bark. Based on the fatty acid composition, the pioneers might decide to leave the tree or enter the bark, feed, and signal to conspecifics by releasing aggregation pheromones. In the *I. typographus* system, we predict that beetles colonizing host trees with high levels of linoleic acid are more likely to get assistance from symbiotic fungi in pheromone production and release.

The fungi *L. abietinum* and *G. europhioides* seemed to have more diverse biosynthetic pathways than the other tested fungi, as they emitted much more spiroacetals when linoleic acid was added to the growth medium. This biochemical versatility might have led to an enhanced symbiotic mutualism with their bark beetle vectors. *Grosmannia europhioides,* a fungus associated with *Ips typographus*, produces the major aggregation pheromone component (2-methyl-3-buten-2-ol) of its bark beetle vector (Zhao et al. 2015b) and here we show that this fungus can do so both in the presence and absence of linoleic acid. The drop in relative amounts of 2-methyl-3-buten-2-ol emitted by *G. europhioides* in linoleic acid-amended medium after day 15 was most likely a consequence of increased emission of *exo*-brevicomin from day 15 and onwards. Because the SPME analysis provides relative amounts of volatiles, an increase in one compound causes a drop in other compounds. As the SPME fiber has different affinity to different compounds SPME analysis will not provide 100% accurate estimates of emitted quantities, but proportional changes in emitted compounds over time will be correct.

We suggest that bark beetles probe the fatty acid composition of tentative host trees to secure fungal assistance with pheromone production and higher nutritional quality for beetle offspring and fungal associates. The chemical communication of bark beetles and their symbiotic fungi deserves further studies. In particular, studies of fungal semiochemical emission in live trees will give new insights into these fascinating tri-partite interactions.

## Supplementary Information

Below is the link to the electronic supplementary material.

Supplementary file1 (DOCX 19.8 KB)

## Data Availability

The datasets generated during and/or analysed during the current study are available from the corresponding author on reasonable request.
