# Supplementary material for: Linoleic Acid Promotes Emission of Bark Beetle Semiochemicals by Fungal Symbionts
Source: J Chem Ecol. 2022 Dec 30;49(1-2):59–66. doi: 10.1007/s10886-022-01400-3 (PMC9941228; doi:10.1007/s10886-022-01400-3)
Supplement: Supplementary file 1 — Supplementary file1 (DOCX 19.8 KB) [file 10886_2022_1400_MOESM1_ESM.docx]

Table S1: Fungal isolates used in this study. All isolates were obtained from the culture collection of the Norwegian Institute of Bioeconomy Research.

| **Fungus** | **Isolate** | | **Collected** |
| --- | --- | --- | --- |
| *Ceratocystiopsis minuta*  (Siemaszko) Upadhyay & Kendrick | Cmin_1 | 1980-93/15 | 1980, Ås, Akershus, Norway |
|  | Cmin_2 | 1981-102/48 | 1981, Ås, Akershus, Norway |
| *Endoconidiophora rufipennis*  (M.J. Wingfield, T.C. Harr & H. Solheim) Z.W. de Beer, T.A. Duong & M.J. Wingfield | Eruf | 1992-633/280/7 | 1992, Caribou Creek, British Columbia, Canada |
| *Grosmannia clavigera*  (Rob. Jeffr. & R.W. Davidson)  Zipfel, Z.W. de Beer | Gcla | 1992-629/122/7 | 1992, Sunday Creek, British Columbia, Canada |
| *Grosmannia europhioides*  (E.F. Wright & Cain) Zipfel, Z.W. de Beer | Geur | 1990-119/20 | 1990, Namsskogan, Nord-Trøndelag, Norway |
| *Leptographium abietinum*  (Peck), M.J. Wingfield | Labi | 1992-632/224/1 | 1992, Williamslake, British Columbia, Canada |
| *Ophiostoma ainoe*  (H. Solheim) | Oain_1 | 1990-60/39 | 1990, Steinkjer, Nord-Trøndelag, Norway |
|  | Oain_2 | 1980-85/37 | 1980, Ås, Akershus, Norway |
| *Ophiostoma bicolor*  Davidson & Wells | Obic | 2004-38/1 | 2004, Ås, Akershus, Norway |
| *Ophiostoma montium*  (Rumbold) Arx | Omon | 1992-628/49/1 | 1992, British Columbia, Canada |
| *Ophiostoma piceae*  (Munch) H. & P. Sydow | Opic | 1980-92/34 | 1980, Ås, Akershus, Norway |
| *Ophiostoma pseudotsugae*  (Peck) M.J. Wingfield | Opse | 1992-634/293/4 | 1992, Wildhorse Creek, British Columbia, Canada |
